# Dysbiosis of Gut Microbiota Is an Independent Risk Factor of Stroke-Associated Pneumonia: A Chinese Pilot Study

**DOI:** 10.3389/fcimb.2021.715475

**Published:** 2021-08-03

**Authors:** Geng-Hong Xia, Ming-Si Zhang, Qi-Heng Wu, Hui-Di Wang, Hong-Wei Zhou, Yan He, Jia Yin

**Affiliations:** ^1^Department of Neurology, Zengcheng Branch of Nanfang Hospital, Southern Medical University, Guangzhou, China; ^2^Department of Neurology, Nanfang Hospital, Southern Medical University, Guangzhou, China; ^3^Microbiome Medicine Center, Department of Laboratory Medicine, Zhujiang Hospital, Southern Medicine University, Guangzhou, China

**Keywords:** gut microbiota, risk, stroke, stroke-associated pneumonia, short-chain fatty acids

## Abstract

**Background and Purpose:**

Identifying risks of stroke-associated pneumonia (SAP) is important for clinical management. We aimed to evaluate the association between gut microbiome composition and SAP in patients with acute ischemic stroke (AIS).

**Methods:**

A prospective observational study was conducted, and 188 AIS patients were enrolled as the training cohort. Fecal and serum samples were collected at admission. SAP was diagnosed by specialized physicians, and disease severity scores were recorded. Fecal samples were subjected to 16S rRNA V4 tag sequencing and analysed with QIIME and LEfSe. Associations between the most relevant taxa and SAP were analysed and validated with an independent cohort. Fecal short-chain fatty acid (SCFA), serum D-lactate (D-LA), intestinal fatty acid-binding protein (iFABP) and lipopolysaccharide binding protein (LBP) levels were measured.

**Results:**

Overall, 52 patients (27.7%) had SAP in the training cohort. The gut microbiome differed between SAP and non-SAP patients; specifically, *Roseburia* depletion and opportunistic pathogen enrichment were noted in SAP patients, as confirmed in the validation cohort (n=144, 28 SAP [19.4%]). Based on multivariate analysis, *Roseburia* was identified as a protective factor against SAP in both cohorts (training, aOR 0.52; 95% CI, 0.30-0.90; validation, aOR 0.44; 95% CI, 0.23-0.85). The combination of these taxa into a microbial dysbiosis index (MDI) revealed that dysbiosis increased nearly 2 times risk of SAP (training, aOR 1.95; 95% CI, 1.19-3.20; validation, aOR 2.22; 95% CI, 1.15-4.26). Lower fecal SCFA levels and higher serum D-LA levels were observed in SAP patients. Furthermore, SAP was an independent risk factor of 30-day death and 90-day unfavorable outcome.

**Conclusion:**

We demonstrate that a microbial community with depleted *Roseburia* and enriched opportunistic pathogens is associated with increased risk of SAP among AIS patients. Gut microbiota screening might be useful for identifying patients at high risk for SAP and provide clues for stroke treatment.

## Introduction

China faces the greatest stroke burden worldwide. According to the recent ‘China Stroke Statistics 2019’ report, stroke was the third most common cause of death (149.49 per 100,000) in China and the leading cause of all-age disability-adjusted life years in 2017. Among those experiencing stroke, ischemic stroke accounted for 81.9% of events (Wang et al., 2020). Medical and neurological complications following stroke are the major cause of mortality in patients with stroke (Patel et al., 2020). Stroke-associated pneumonia (SAP) is the leading complication after stroke and is reported to lead to poor stroke outcomes and longer hospital stays and increase the occurrence of severe disability and even mortality (Hannawi et al., 2013; Li et al., 2014; Nam et al., 2018; Patel et al., 2020). Despite the clinical importance of SAP, the low sensitivity of chest X-rays and sputum cultures and the atypical and vague clinical manifestations make SAP difficult to diagnose (Hassan et al., 2006; Li et al., 2014; Kishore et al., 2015). Risk factors for SAP, including age, stroke severity (assessed with the National Institutes of Health stroke scale, NIHSS), atrial fibrillation (AF), dysphagia, ventilator use and prolonged hospital stay, have been studied (Sui and Zhang, 2011; Hannawi et al., 2013; Li et al., 2014; Patel et al., 2020), and several clinical predictive models for SAP have been validated and used to select patients at high risk of SAP (Hoffmann et al., 2012; Ji et al., 2013). However, the incidence of SAP remains high, with an incidence of 10% to 32% in patients with stroke in China (Wang et al., 2020). Additionally, prevention of SAP has not advanced substantially (Westendorp et al., 2015). Thus, there is an urgent need to expand our knowledge of the as-yet undetermined factors that are associated with or even causative of SAP.

The gut microbiota plays a critical role in the pathogenesis of lung inflammation (Stanley et al., 2016) as well as the lung infection that occurs in stroke (Mjösberg and Rao, 2018). In a mouse model of stroke, SAP occurred in specific pathogen-free mice but not germ-free mice. Stroke-induced gut barrier permeability and dysfunction has been shown to precede the dissemination of orally inoculated bacteria into peripheral tissues (Mjösberg and Rao, 2018). Mice with gut microbiota depletion induced by antibiotics exhibit increased bacterial dissemination, inflammation and mortality, and their alveolar macrophages show a diminished capacity to clear pathogens from the lungs. Normalization of the gut microbiota can rescue defects in the bactericidal effects of alveolar macrophages (Schuijt et al., 2016; Brown et al., 2017). Additionally, the gut microbiota and metabolites (short-chain fatty acids [SCFAs]) were reported to protect the host against pneumonia *via* multiple immune mechanisms (Schuijt et al., 2016; Brown et al., 2017; Antunes et al., 2019), including GM-CSF (Brown et al., 2017) and the GPR43-type 1 interferon response (Antunes et al., 2019). These results show the close relationship between the gut microbiota and pneumonia in stroke.

The clinical importance of the gut microbiota in SAP is only beginning to be understood. In a relatively small patient cohort, the majority of the microorganisms detected in 8 patients with SAP were common commensal bacteria that normally reside in the intestinal tract (Mjösberg and Rao, 2018). A recent clinical study from the Netherlands, with 14 patients who developed infection (4 pneumonia cases) among 349 patients with ischemic and hemorrhagic stroke, demonstrated that a lower abundance of butyrate-producing bacteria was associated with stroke and stroke-associated infections (Haak et al., 2020). However, the very limited numbers of SAP subjects in these studies made it difficult to generate statistically significant conclusions. Furthermore, clinical data concerning the association between the gut microbiota and SAP in China remain limited. We previously reported that disruption of the gut microbiota and its metabolites, including trimethylamine-N-oxide and SCFAs, are associated with stroke injury and outcome in AIS patients (Xia et al., 2019; Tan et al., 2020; Xu et al., 2021). We further identified that an expansion of *Enterobacteriaceae* in the gut is an independent risk factor for poor functional outcome in AIS patients (Xu et al., 2021). In the present study, we conducted two independent cohorts to investigate whether the gut microbiota composition correlates with and predicts SAP in patients with AIS.

## Materials and Methods

### Patient Cohort and Clinical Data Collection

**Figure 1:**
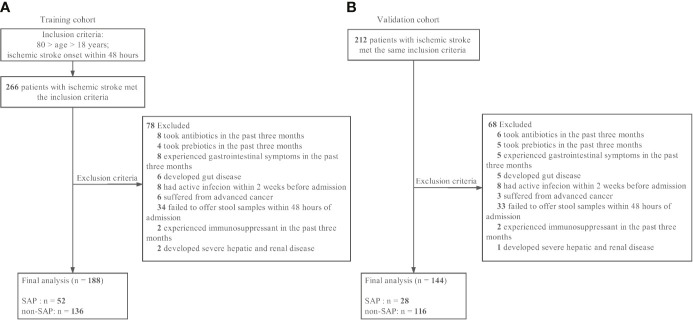
Patient flowcharts. **(A)** The training cohort. **(B)** The validation cohort. SAP indicates stroke-associated pneumonia.

The training patient cohort was enrolled in the Department of Neurology at South China’s Nanfang Hospital between June 2017 and December 2018. Patients with acute ischemic stroke within 48 hours of stroke onset and met the inclusion criteria were considered recruited in this study. Ischemic stroke was defined as a clinical syndrome associated with a radiographically proven acute infarct consistent with a vascular pattern with supported brain imaging—magnetic resonance imaging or magnetic resonance angiography. Diagnosis of ischemic stroke was confirmed for all participants at admission for the index stroke. The detailed enrollment process is shown in **Figure 1**. Inclusion criteria were as follows: (1) age > 18 years, (2) admission within 48 hours of ischemic stroke onset. Exclusion criteria were as follows: (1) antibiotics, prebiotics, or probiotics used within three month before admission; (2) admission after 48 hours of stroke onset; (3) experienced gastrointestinal disease symptoms in the past three months; (4) developed gut diseases; (5) active infection within the 2 weeks before admission; (6) suffered from advanced cancer; (7) failed to offer stool samples within 48 hours of admission; (8) history of systemic disease such as cirrhosis, renal failure and hematologic disease or use of an immunosuppressant. An independent cohort including AIS patients recruited from the same hospital from February 2014 and December 2015, were used for validation using the same selection criteria of training cohort patients and fecal samples were collected within 48 hours of admission. A total of 212 AIS patients met the same inclusion criteria and 144 AIS patients were finally recruited as the validation cohort as 68 patients were excluded according to the exclusion criteria. Details of the enrollment process are shown in **Figure 1**. The Ethical Committee of Southern Medical University approved all aspects of this study, and informed consent for data collection was obtained from all subjects or their legal guardians. Patient’s demographics and clinical characteristics were obtained at the time of admission. Fecal samples were obtained within 48 hours of admission and frozen at -80°C within 2 hours of collection.

Demographic (age, sex), vascular risk factors (hypertension, hyperlipidemia, atrial fibrillation (AF), diabetes mellitus (DM), current smoking, and previous stroke history) and clinical factors (initial stroke severity, dysphagia, the use of thrombolytic therapy and stroke subtype) were assessed and recorded. Stroke subtypes were classified according to the Trial of Org 10172 in Acute Stroke Treatment classification. A2DS2 score, a well-validated tool for predicting SAP, ranging from 0 to 10 and combines age (1 point for ≥75 years), atrial fibrillation (1 point), dysphagia (2 points), sex (1 point for male sex), and initial stroke severity (0 point for NIHSS score 0–4, 3 points for NIHSS score 5–15, and 5 points for NIHSS score ≥16) was also assessed for a sensitivity analysis (Vyas et al., 2019).

This study was approved by the Ethics Committee of Nanfang Hospital, Southern Medical University (NFEC-2016-148), and registered at http://www.chictr.org (ChiCTR-ROC-17011567).

### Definition of Stroke-Associated Pneumonia (SAP) and Long-Term Functional Outcome

Our primary outcomes of interest were predictors of stroke-associated pneumonia (SAP) following AIS, which were recorded as patients developed lower respiratory tract infections according to the modified Centers for Disease Control and Prevention criteria during the first 7 days after the stroke onset (Smith et al., 2015). Consensus of diagnosis criteria of SAP was reached for the following: (1) stroke-associated pneumonia (SAP) is the recommended terminology for the spectrum of lower respiratory tract infections within the first 7 days after stroke onset; (2) modified Centers for Disease Control and Prevention (CDC) criteria are proposed for SAP as follows-probable SAP: CDC criteria met, but typical chest x-ray changes absent even after repeat or serial chest x-ray; definite SAP: CDC criteria met, including typical chest x-ray changes; (3) there is limited evidence for a diagnostic role of white blood cell count or C-reactive protein in SAP; and (4) there is insufficient evidence for the use of other biomarkers (e.g., procalcitonin) (Smith et al., 2015). The treating physician diagnosed the SAP during the hospitalization, according to the criteria mentioned above. Treating physicians were blinded to the other clinical and laboratory findings regarding the primary diagnosis, as well as the secondary validation. The onset of clinical symptoms led to further investigations and resulted in the diagnosis of SAP. In the current study, both probable and definite SAP as determined by the modified Centers for Disease Control criteria were equally coded as SAP, excluding the burden of chest x-ray findings (Nam et al., 2017). Detail Pneumonia Severity Index (PSI), a well-validated scoring system of pneumonia burdens in participants with SAP (Shah et al., 2010), was used to assess the burden or severity of SAP. The quick Sepsis-related Organ Failure Assessment (qSOFA) score was used to evaluate the sepsis risk in SAP patients; patients with qSOFA scores ≥2 were likely to have sepsis (Seymour et al., 2016). Patients who had ≥IV class (PSI score >90) PSI scores (high PSI group) or qSOFA scores ≥2 were considered to have severe SAP (Shah et al., 2010; Nam et al., 2017).

To evaluate the impact of SAP on stroke outcomes, we assessed the clinical outcome in the training cohort including hospitalization duration, discharge NIHSS score, 30-day mortality and 3-month modified Rankin Scale (3 m-mRS) scores by reviewing the medical records during hospitalization, the medical records from outpatient clinics or followed-up by telephone for patients or their caregivers.

### Microbiological Investigation of Fecal Samples

We used QIIME (1.9.1) for the following analyses. All samples were normalized to 8000 sequences to avoid deviation caused by the effects of different sequencing depths. The UniFrac distance was applied to analyze beta diversity (Seymour et al., 2016). The principle coordinate analysis (PCoA) is a dimensionality reduction method illustrating the relationship between samples based on a distance matrix. PCoA could be used to visualize the unsupervised grouping pattern of a complex data set such as a microbiome. Chosen information related to a microbiome can be shown as either a clear separation or a trend in PCoA by coloring samples. The linear discriminant analysis effect size (LEfSe) was applied to determine differential taxa between groups (Lozupone et al., 2011). LEfSe is an algorithm for high dimensional biomarker discovery that can identify metagenomic features characterizing differences between two or more biological conditions. After coupling standard tests for statistical significance with additional tests encoding biological consistency and effect size, features that were most likely to explain the differences between the classes were determined by LEfSe analysis (Lozupone et al., 2011). The linear discriminant analysis (LDA) threshold was set at 4 in the training cohort. The LDA score was calculated for each of the differential features detected by LEfSe, and a higher score represented greater differences in features between the tested groups. Phylogenetic Investigation of Communities by Reconstruction of Unobserved States (PICRUSt) algorithm was performed in QIIME to predict the functional profiles of the bacterial metagenomes (Kyoto Encyclopedia of Genes and Genomes, KEGG) in the two groups based on the relative abundance of individual OTUs. We calculate the log of [total abundance in organisms increased in SAP] over [total abundance of organisms decreased in SAP] for all samples, hereafter referred to as the Microbial Dysbiosis index (MDI) (Segata et al., 2011). The original data used in the present study were uploaded into the BioProject database (http://www.ncbi.nlm.nih.gov/bioproject/746092) at accession number PRJNA746092.

### Fecal SCFA Detection

SCFA was analyzed in the training cohort. 0.2 g of each fecal sample was separated. Six analytes were targeted for SCFA analysis including acetic acid (Dr. Ehrenstorfer, Germany), propionic acid (Dr. Ehrenstorfer, Germany), isobutyric acid (Supelco, United States), butyric acid (Dr. Ehrenstorfer, Germany), isovaleric acid (Sigma-Aldrich, United States), and valeric acid (Nu-Chek, United States). fecal samples were homogenized in 1.0 mL of ultrapure water that contained an internal standard, 2,2-dimethylbutyric acid (Dr. Ehrenstorfer, Germany). The supernatant was transferred into a new tube after centrifugation, followed 10 µL of 50% sulfuric acid and 0.5 g of sodium sulfate (Macklin, China) were added to the tube along with analytically pure diethyl ether (2 mL). The mixture was vortexed for 1 minute and then centrifuged at 5000 rpm (room temperature, 10 minutes). The ether layer was finally collected for gas chromatography with mass selective detection (5977B GC/MSD, Agilent Technologies, Santa Clara, CA, United States) measurement. The Gas chromatography mass spectrometry (GC/MS) data were acquired and analyzed using MassHunter Workstation software (Agilent Technologies) running on Windows 7 (Microsoft, Redmond, WA, United States). Final concentrations were calculated based on internal standards and are expressed as micromoles per gram of wet feces (μmol/g).

### Quantification of Serum iFABP, D-Lactate and LBP

Serum samples were collected at admission and isolated by centrifugation at 3000 rpm for 10 minutes and stored at −80°C until testing. Concentrations of intestinal fatty acid–binding protein (iFABP), D-lactate (D-LA) and lipopolysaccharide-binding protein (LBP) levels were determined using commercially available enzyme-linked immunosorbent assay kits (Bioswamp, Myhalic Biotechnology Co, Ltd, Wuhan, China). Standard curves were all within the expected range and all measurements were performed by one experienced staff blinded to the study design.

### Statistical Analysis

All statistical analyses were performed using SPSS version 24 (IBM SPSS, Chicago, IL). The results are expressed as numbers (percentages, %) for categorical variables and medians (interquartile ranges) for continuous variables. We transformed continuous variables with skewed data into a log scale. Mann-Whitney U-test was used for continuous variables and the χ2 test and Fisher exact test were used for categorical variables. Variables including age, current smoking, dysphagia, atrial fibrillation, initial NIHSS score, white blood cell (WBC), neutrophils (NEU), neutrophil-to-lymphocyte ratio (NLR) and history of stroke, hyper blood pressure (HBP), diabetes mellitus (DM) and hyperlipidemia (HLP), potential confounding factors previously reported to be associated with SAP (Westendorp et al., 2015; Haak et al., 2020) were analysed. All variables showing a trend in association in univariate analysis (P<0.10) were included in the multivariable model. The variables entered in the multivariate logistic regression model were as follows: age, dysphagia, atrial fibrillation, initial NIHSS score, WBC, NEU, NLR and history of HLP. Predictive performance for SAP was assessed by comparing receiver operator characteristic (ROC) curves. The relative risk was expressed as the odds ratio (OR) with the 95% confidence interval (CI).

## Results

### Baseline Characteristics

According to the inclusion and exclusion criteria, 188 eligible AIS patients were recruited as the training cohort. Details of the enrollment process are shown in **Figure 1**. The median initial NIHSS score at admission was 4 [2.0–10.25], and 66.5% of the subjects were male. Among them, 52 subjects (27.6%) developed overt clinical pneumonia within 7 days after stroke onset (SAP). The baseline characteristics between groups with and without SAP are presented in **Table 1**. The SAP group was older and had higher rates of hyperlipidemia, AF, cardioembolic stroke, dysphagia and initial thrombolytic therapy and lower rates of small vascular occlusion than the non-SAP group. The SAP group also had higher initial NIHSS and A2DS2 scores, white blood cell (WBC) counts, neutrophil (NEU) counts, neutrophil-lymphocyte ratios (NLR) and CRP levels and lower lymphocyte (LYM) counts.

**Table 1:** Baseline characteristics of patients with SAP and Non-SAP with acute ischemic stroke.

	Training cohort (n=188)		
	Non-SAP (n=136)	SAP (n=52)	P value
Age, y, mean (SD)	57.7 (13.5)	64.5 (14.2)	0.002
Sex, male (%)	86 (63.2)	39 (75.0)	0.126
Hypertension (%)	75 (55.1)	33 (63.5)	0.302
Diabetes mellitus (%)	32 (23.5)	9 (17.3)	0.355
Hyperlipidemia (%)	6 (4.4)	7 (13.5)	0.029
Atrial fibrillation (%)	6 (4.4)	22 (42.3)	<0.001
Smoking (%)	54 (39.7)	19 (36.5)	0.690
Stroke history (%)	24 (17.6)	8 (15.4)	0.712
Intial NIHSS score [IQR]	3 [1–6]	13 [9–18]	<0.001
Dysphagia (%)	26 (19.1)	46 (88.5)	<0.001
A2DS2 score [IQR]	1 [1–4]	6 [5–7]	<0.001
Thrombolysis (%)			<0.001
None	111 (81.6)	26 (50.0)	
Intravenous	10 (7.4)	2 (3.8)	
Intra-arterial	5 (3.7)	5 (9.6)	
Both	10 (7.4)	19 (36.5)	
Stroke subtype (%)			<0.001
Large artery disease	61 (44.9)	30 (57.7)	
Small vessel occlusion	41 (30.1)	1 (1.9)	
Cardioembolism	15 (11.0)	14 (26.9)	
Other determined	5 (3.7)	1 (1.9)	
Undetermined	14 (10.3)	4 (7.7)	
WBC, × 10^9^/L	8.15 [6.43–9.54]	9.88 [8.39–13.38]	<0.001
NEU, × 10^9^/L	5.11 [3.91–6.82]	8.01 [6.20–10.95]	<0.001
LYM,× 10^9^/L	1.81 [1.42-2.41]	1.21 [0.93-1.71]	<0.001
NLR	2.91 [1.86-4.20]	7.13 [4.07-11.28]	<0.001
CRP	2.25 [1.09-6.51]	22.67 [8.68-61.41]	<0.001

SD, standard deviation; IQR, interquartile range; NIHSS, National Institutes of Health Stroke Scale; SAP, stroke-associated pneumonia. WBC, white blood cell count; NEU, neutrophil count; LYM, lymphocyte count; NLR, neutrophil-to-lymphocyte rate; CRP, C-reactive protein.

### The Gut Microbiome Profile Differs the Two Groups, With *Roseburia* Depletion and Opportunistic Pathogen Enrichment in the SAP Group

**Figure 2:**
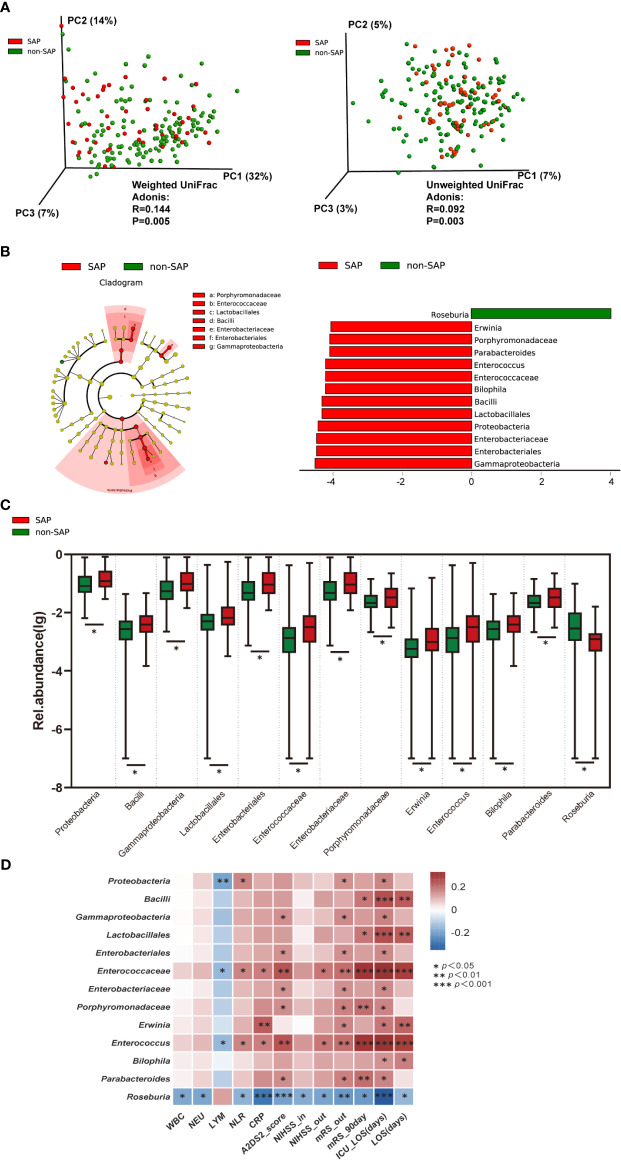
Comparison of the microbial communities of the SAP and non-SAP groups in the training cohort. **(A)** PCoA plot with Weighted and Unweighted UniFrac distance analyses demonstrating that the bacterial communities were significantly different between the SAP (n = 52) and non-SAP group (n = 136). **(B)** LEfSe identified the 13 most differentially abundant taxa with relative abundance higher than 0.1% between the two groups in the training cohort. **(C)** Relative abundance of the 13 taxa differed in two patient groups. **(D)** Heat map of Spearman’s rank correlation coefficient among clinical indexes and 13 taxa. NIHSS-in, initial NIHSS score; mRS, modified Rankin Scale. NIHSS/mRS-out, discharge NIHSS/mRS score. A2DS2, includes age (1 point for ≥75 years), atrial fibrillation (1 point), dysphagia (2 points), male sex (1 point), and stroke severity (0 point for NIHSS score 0–4, 3 points for NIHSS score 5–15, and 5 points for NIHSS score ≥16).

To determine whether the microbiome structures of the patients with and without SAP were significantly different, we used principal coordinate analysis (PCoA). Based on the weighted UniFrac distance and the unweighted UniFrac distance, the microbial composition in SAP patients was significantly different from that in patients without SAP (Adonis test, weighted, R = 0.144, P = 0.005; unweighted, R = 0.092, P = 0.003) (**Figure 2A**). These results remained significant based on other distances, including Bray-Curtis (R = 0.129, P = 0.001) and Pearson (R = 0.156, P = 0.001) distances (**Figures S1A, B**). Next, we conducted analysis with LEfSe, an algorithm for high-dimensional biomarker discovery (Segata et al., 2011), to identify the most relevant taxa responsible for the observed differences between the SAP and non-SAP groups. This analysis identified 13 taxa that were differentially abundant in the two patient groups (**Figure 2B**), and the relative abundances of these taxa differed significantly between the SAP and non-SAP groups (**Figure 2C**). Compared to patients without SAP, *Roseburia* was identified as the most relevant taxon that was significantly depleted in SAP patients, which showed inverse correlations with CRP level (r = - 0.285, P = 0.0004), discharge and 90-day mRS scores (r = - 0.211, P = 0.004; and r = - 0.207, P = 0.004, respectively), and ICU length of stay (ICU-LOS, r = - 0.344, P < 0.001) (**Figure 2D**). Another 12 taxa were enriched in SAP (**Table 2**), mostly representing opportunistic pathogens, including five *Proteobacteria* taxa, class *Gammaproteobacteria*, order *Enterobacteriales*, family *Enterobacteriaceae*, the genera *Erwinia* and *Bilophila*; and two *Bacteroidetes* taxa, family *Porphyromondaceae* and genus *Parabacteroides*; and four *Firmicutes* taxa, class *Bacilli*, order *Lactobacillales*, family *Enterococaceae* and genus *Enterococcus*, were enriched in SAP compared to those without SAP (**Figure 2D**). Some SAP-enriched taxa, including *Enterococcus*, was correlated positively with discharge and 90-day mRS scores (r = 0.200, P = 0.006; r = 0.297, P < 0.001; respectively) and ICU-LOS (r = 0.331, P < 0.0001) (**Figure 2D**).

**Table 2:** Relative abundance of selected microbial taxa in patients with and without SAP in the training cohort.

Taxa	Relative abundance (%)			Univariate		Multivariate	
The taxa increased in SAP	SAP	non-SAP	P value	OR (95% CI)	AUC	Model 1*aOR (95% CI)	Model 2†aOR (95% CI)
Phylum *Proteobacteria*	12.04	8.19	0.008	3.00 (1.28-7.00)	0.624	NA	NA
Class *Gammaproteobacrteria*	9.60	5.37	0.003	2.91 (1.44-5.85)	0.641	NA	3.79 (1.19-12.13)
Class *Bacilli*	0.67	0.51	0.011	2.20 (1.27-3.83)	0.620	2.65 (1.08-6.49)	2.80 (1.19-6.56)
Order *Lactobacillales*	0.67	0.51	0.011	2.20 (1.27-3.83)	0.620	2.65 (1.08-6.49)	2.80 (1.19-6.56)
Order *Enterobacteriales*	9.22	4.77	0.004	2.66 (1.35-5.25)	0.635	NA	3.78 (1.19-11.95)
Family *Enterococcaceae*	0.32	0.14	<0.001	2.35 (1.44-3.86)	0.701	2.58 (1.27-5.25)	1.98 (1.10-3.60)
Family *Porphyromonadaceae*	3.31	2.16	0.023	2.63 (1.09-6.36)	0.607	NA	NA
Family *Enterobacteriaceae*	9.22	4.77	0.004	2.66 (1.35-5.25)	0.635	NA	3.78 (1.19-11.95)
Genus *Erwinia*	0.099	0.057	0.003	1.32 (0.97-1.80)	0.642	2.08 (1.23-3.51)	2.07 (1.25-3.43)
Genus *Enteococcus*	0.324	0.135	<0.001	2.35 (1.44-3.86)	0.701	2.58 (1.27-5.25)	1.98 (1.10-3.60)
Genus *Bilophila*	0.387	0.274	0.010	2.64 (1.30-5.38)	0.622	3.75 (1.22-11.57)	4.94 (1.36-17.92)
Genus *Parabacteroides*	3.31	2.16	0.023	2.63 (1.09-6.36)	0.607	NA	NA
The taxa decreased in SAP							
Genus *Roseburia*	0.13	0.29	<0.001	0.50 (0.33-0.78)	0.701	0.52 (0.30-0.90)	0.56 (0.34-0.93)
Microbial Dysbiosis Index (MDI)‡	2.6	2.1	<0.001	2.17 (1.45-3.25)	0.750	1.95 (1.19-3.20)	1.90 (1.18-3.04)

*Adjusted with P <0.10 in univariate analysis (age, hyperlipidemia, AF, dysphagia, white blood cell, neutrophil, lymphocyte, NLR, TOAST and initial NIHSS score).

†Adjusted with P<0.10 in the univariate analysis and A2DS2 (hyperlipidemia, white blood cell, neutrophil, lymphocyte, NLR, TOAST and A2DS2 score).

‡Index of Microbial dysbiosis (showed as median), not relative abundance.

NA, not assessed.

To validate enrichment and depletion of taxa in SAP, an independent cohort of 144 AIS patients was recruited according to the inclusion and exclusion criteria (median admission NIHSS score, 4 [2-6]; male, 75.5%; SAP group n=28 [19.4%]; non-SAP group n=116 [80.6%]) (**Figure 1** and **Table S1**). The characteristics of the gut microbiota in patients with SAP were significantly different from those in non-SAP subjects, as indicated by PCoA (**Figure S2A**). As indicated by LEfSe analysis (**Figure S2B**), we could detect statistically significant depletion of *Roseburia* and enrichment in *Proteobacteria*, *Gammaproteobacteria*, *Enterobacteriales*, *Enterobact*eriace*ae*, *Bacilli* and *Lactobacillales* in subjects with SAP compared to those without SAP. Additionally, *Enterococcaceae* and *Enterococcus* reads were enriched in SAP patients from the validation cohort (**Table S2**). These results were consistent with the results obtained in the training cohort.

Since age, stroke severity and dysphagia were established risk factors of SAP (Hoffmann et al., 2017), and the great discrepancies were observed in age, stroke severity and dysphagia between patients with and without SAP in our series (see **Table 1** and **Table S1**), we further addressed whether the gut microbial profile was correlated to these factors. Overall, increasing age (**Figure S3A**) and NIHSS (**Figure S3B**), as well as dysphagia (**Figure S3C**), could differentiate the gut microbiota profiles of the full sample set in the training cohort. However, when we performed age-, NIHSS- and dysphagia- matched comparisons of the gut microbiota in patients with and without SAP, including 25 dysphagic patients with SAP (mean age 62 years; median NIHSS score 11) and twenty-five age- and NIHSS- matched dysphagic non-SAP subjects (mean age 61 years; median NIHSS score 8) (**Table S3**), we observed statistically significant differences in the Bray-Curtis and Pearson distances (**Figures S4A, B**), reinforcing that the gut microbiota composition is different in SAP and non-SAP settings. Also in the age-, NIHSS- and dysphagia- matched comparisons, depleted *Roseburia* and enriched opportunistic pathogen (i.e., *Enterococcaceae*, *Enterococcus*, *Erwinia*) were observed in SAP patients (**Figures S4C, D**).

Phylogenetic Investigation of Communities by Reconstruction of Unobserved States (PICRUSt) analysis was performed to infer microbial metabolites and metabolic pathways (**Figure S1C**). As shown in the results, pathways correlated with replication and repair and the immune system were significantly depleted in the SAP group.

### Microbial Dysbiosis Is Associated With SAP

**Figure 3:**
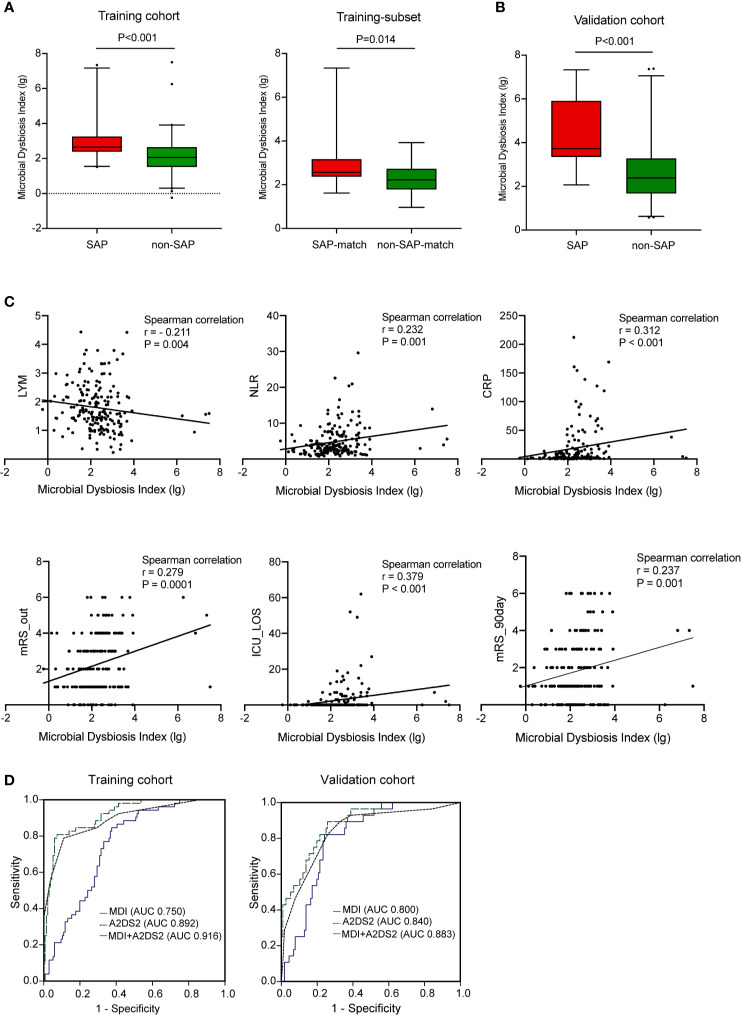
Microbial dysbiosis is associated with SAP. **(A)** Box plot showing the MDI in the SAP and non-SAP groups in all patients (**A**, left) and age- and NIHSS- matched subset (**A**, right) in the training cohort. **(B)** Box plot showing the MDI of the validation cohort. **(C)** Correlation between MDI and clinical parameters including markers of immunosuppression (LYM), system inflammation (NLR, CRP), and outcomes (ICU-LOS, discharged and 90 days- mRS score). **(D)** Predictive performance of MDI, A2DS2 and the predictive model (A2DS2 plus MDI) for SAP in the training cohort (**D**, left) and in the validation cohort (**D**, right). Training cohort (n=188); Validation cohort (n=144). AUC, area under the curve; MDI, microbial dysbiosis index; ROC, receiver operating characteristic.

Next, we combined 13 of the most relevant taxa that characterized each group of patients and calculated the microbial dysbiosis index (MDI) (Ferreira et al., 2018). The intestinal microbiota of patients with SAP exhibited a higher MDI value than that of patients without SAP in both the training cohort (P<0.0001; **Figure 3A**, left) and the validation cohort (P<0.0001; **Figure 3B**). Similar findings were observed in the age-, NIHSS- and dysphagia- matched subset of the training cohort (**Figure 3A**, right). The MDI value was negatively related to the LYM count (r = - 0.211, P = 0.004) and positively related to the NLR (r = 0.232, P = 0.001), the CRP level (r = 0.312, P < 0.001), biomarkers of immunosuppression and inflammation (Suzuki et al., 2018), ICU-LOS (r = 0.379, P < 0.001), and discharge and 90-day mRS scores (r = 0.279, P = 0.0001; and r = 0.237, P = 0.001, respectively) (**Figure 3C**). We further assessed the relationships between pneumonia severity and MDI. Interestingly, the high PSI group (PSI>90, n=33) had a significantly higher MDI than the low PSI group (n=19) (**Figure S5A**, left). These results remained significant in the high qSOFA group (qSOFA ≥2, n=20) compared to the low qSOFA group (n=32) (**Figure S5A**, right). These results demonstrated that this dysbiosis was associated with immunosuppression, systemic inflammation, pneumonia severity and poor clinical outcomes in AIS patients.

In multivariable logistic regression adjusted for age, NIHSS score, dysphagia, AF, hyperlipidaemia, WBC counts, NEU counts and NLR, the MDI was an independent risk factor for SAP in both the training (aOR 1.95, 95% CI [1.19-3.20]) (**Table 2**) and validation (aOR 2.22, 95% CI [1.15-4.26]) cohorts (**Table S2**). In terms of the specific microbial taxa, *Roseburia* was a protective factor against SAP (training, aOR 0.52, 95% CI [0.30- 0.90]; validation, aOR 0.44, 95% CI [0.23- 0.85]). These results remained consistent after we used the A2DS2 score to replace age, AF, initial NIHSS score, and dysphagia for sensitivity analysis.

We also evaluated whether the MDI could be used to discriminate between patients in the SAP and non-SAP groups. In receiver operating characteristic (ROC) analysis, the MDI showed moderate-to-high performance in identifying SAP, yielding areas under the curve (AUCs) of 0.750 and 0.800 for the training and validation cohorts, respectively. The AUC of the A2DS2 score, the clinical predictive model for SAP (Vyas et al., 2019), was increased upon combination with the MDI for the training (0.892 to 0.916) and validation cohorts (0.840 to 0.883) (all P<0.05, **Figure 3D**). The MDI exhibited improved sensitivity and specificity to detect SAP when compared with the use of a single taxon (**Table 2**).

### Decreased Fecal SCFA Levels and Increased Gut Permeability in SAP Patients

**Figure 4:**
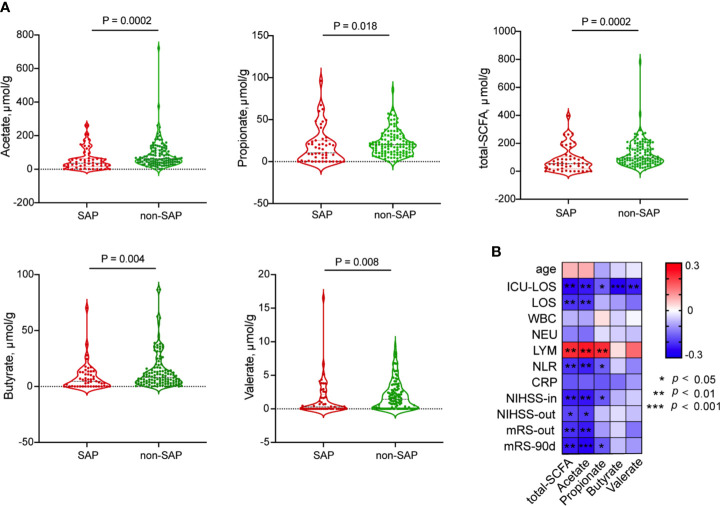
Comparison of fecal SCFAs levels between patients with SAP and without SAP. **(A)** Decreased fecal SCFA, including acetate; propionate; butyrate and valerate, were in the SAP group than in the non-SAP group. **(B)** Spearman’s Correlation of SCFAs with clinical parameters.

Because *Roseburia*, the most abundant SCFA-producing bacteria, was consistently depleted in the gut microbiota of patients with SAP, we further evaluated the concentrations of fecal SCFAs, including acetate, propionate, butyrate and valerate, in patients with and without SAP. The concentrations of acetate, propionate, butyrate and valerate decreased significantly in the fecal samples from patients with SAP (median [IQR], acetate: 34.54 [15.30-63.35] µmol/g; propionate: 10.35 [0.46-25.15] µmol/g; butyrate: 4.74 [0.00-12.84] µmol/g; valerate: 0.28 [0.00-2.12] µmol/g) compared with those in samples from patients without SAP (median [IQR], acetate: 63.09 [39.78-115.27] µmol/g; propionate: 20.12 [10.80-29.99] µmol/g; butyrate: 8.25 [3.43-16.39] µmol/g; valerate: 1.43 [0.17-2.90] µmol/g) (**Figure 4A**). These SCFAs were mostly negatively related to the ICU-LOS (acetate, r = - 0.230; propionate, r = - 0.160; butyrate, r = - 0.257; valerate, r = - 0.228) (**Figure 4B**). We further assessed the relationships between pneumonia severity and *Roseburia* or SCFAs levels. Interestingly, the high PSI group (PSI>90, n=33) had a significantly lower abundance of gut *Roseburia* and lower butyrate content than the low PSI group (n=19) (**Figures S5A, C**). These results remained significant in the comparison of the high qSOFA group (qSOFA ≥2, n=20) and low qSOFA group (n=32) (**Figures S5B, D**).

**Figure 5:**
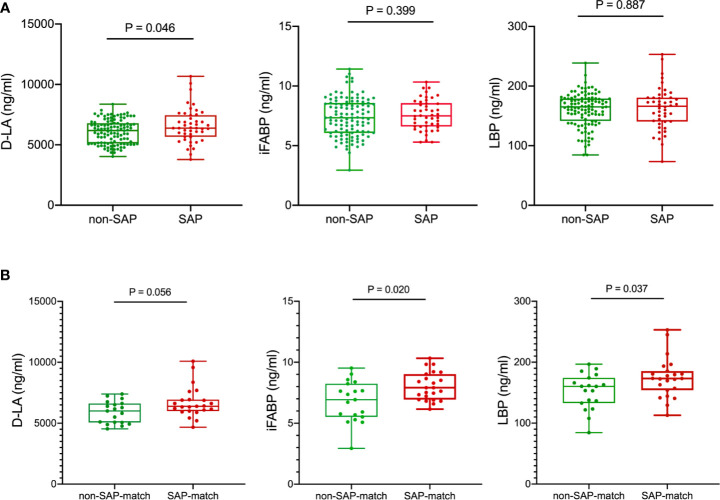
Comparison of serum intestinal integrity biomarkers between patients with SAP and without SAP in the training cohort **(A)** and age-, NIHSS- and dysphagia- matched subset **(B)** of the training cohort. Patients with SAP had increased serum D-LA, iFABP and LBP levels at the early acute stage than patients without SAP. Mann-Whitney *U* test. D-LA, D-lactate; iFABP, intestinal fatty acid-binding protein; LBP, lipopolysaccharide-binding protein.

Next, we quantified the plasma concentrations of D-lactate (D-LA), intestinal fatty acid–binding protein (iFABP) and lipopolysaccharide-binding protein (LBP) (**Figure S6**), which were previously reported as intestinal integrity biomarkers (Camara-Lemarroy et al., 2020), to evaluate intestinal permeability in patients with and without SAP. The concentrations of D-LA, a marker of intestinal bacterial translocation (Camara-Lemarroy et al., 2020), were significantly increased in plasma samples from patients with SAP (mean ± SD, 6588.1 ± 1417.9 ng/mL) compared with those in plasma from non-SAP subjects (6083.3 ± 983.3 ng/mL) (**Figure 5A**). Also in age-, NIHSS- and dysphagia- matched subset, higher serum D-LA, iFABP and LBP levels were observed in patients with SAP (D-LA, 6710.1 ± 1271.7 ng/mL; iFABP, 8.021 ± 1.154 ng/mL; LBP, 173.3 [154.1-185.4] ng/mL) compared to those without SAP (D-LA, 5898.0 ± 903.7 ng/mL; iFABP, 6.787 ± 1.672 ng/mL; LBP, 160.2 [133.0-174.2] ng/mL) (**Figure 5B**), indicating that intestinal mucosal integrity was significantly reduced in patients who further developed a clinically overt pneumonia.

### Poor Functional Outcome in SAP Patients

The clinical outcomes between groups with and without SAP are presented in **Table S4**. The SAP group showed a longer duration of hospitalization (15.5 [12.0–22.0] days versus 9.0 [7.0–12.0] days; P<0.001), higher discharge NIHSS score (9.5 [2.5–13.0] versus 2.0 [0–4.0]; P<0.001), higher rates of 30-day mortality (17.3% versus 1.5%; P<0.001) and GH events (25.0% versus 3.7%; P<0.001) and more severe 90-day mRS score (mRS ≥3, 73.1% versus 12.5%; P<0.001). After adjustment for age and NIHSS score, SAP was an independent risk factor for GH events (aOR 8.73, 95% CI [2.93 - 26.02]), 30-day death (aOR 7.17, 95% CI [1.22 - 42.30]) and 90-day unfavorable outcome (aOR 7.22, 95% CI [2.90 - 17.97]).

## Discussion

In this study, we found that the gut microbiota composition in patients with SAP at the early acute stage was different from that in patients without SAP. Microbial community dysbiosis, characterized by depleted *Roseburia* and enriched opportunistic pathogen, was associated with increased risk of SAP and pneumonia severity among AIS patients. Addition of this dysbiosis increased the predictive performance of clinical models of SAP. Decreased fecal SCFA levels and increased serum D-LA levels were noted in patients with SAP compared to those in patients without SAP. The major findings regarding the gut microbial profiles revealed in the training cohort were confirmed in an additional validation cohort.

In contrast to previous studies concerning the traditional risk factors for SAP (Sui and Zhang, 2011; Hannawi et al., 2013; Li et al., 2014; Patel et al., 2020), this study considered the dysbiotic gut microbiota in stroke patients at the early acute stage as a potential risk factor for SAP. As a common medical complication after stroke, stroke-associated pneumonia (SAP) is reported to affect 7% to 38% of patients with stroke (Nam et al., 2018). Stroke severity is a well-known risk factor for SAP and patients with SAP had significantly higher NIHSS score at admission in our study (median NIHSS, 13), which was similar to previous data reported by Nam KW et al. (median NIHSS, 13) (Nam et al., 2018). The reason for higher SAP rate in our study is that more patients with moderate-to-severe stroke were admitted in our hospital, which tend to receive patients with moderate-to-severe stroke because of the triage policy of the Chinese Stroke Network. This allowed us to investigate the role of dysbiotic gut microbiota on SAP *via* the relative large patient data. Concerning the heterogeneity of the ischemic stroke population, we recruited two patient cohorts to assure stably different taxa between patients with and without SAP. In our study, the gut microbial communities in the SAP and non-SAP groups were structurally different, which were characterized by using culture-independent techniques, illustrated by a PCoA plot, and validated by an additional independent cohort. These dissimilarities in gut microbial profiles remained significant after adjustment for age, stroke severity and dysphagia. In terms of the composition of the gut microbiota, we were able to determine the bacterial taxa that most likely explain the differences between the SAP and non-SAP groups by applying the LEfSe algorithm, which has been validated for high-dimensional microbiome data sets. Some disease-promoting pathogens, such as *Enterococcus* (Xia et al., 2019; Tan et al., 2020; Xu et al., 2021), overgrew in SAP subjects and were related to worse clinical outcomes in these patients. The integration of data regarding the most relevant taxa that characterized each patient group allowed us to calculate the MDI, which was significantly elevated in patients with SAP, especially in those with severe pneumonia, and related to systemic inflammation and immunosuppression, two important variables of SAP (Hoffmann et al., 2017). Functional profiling of microbial communities obtained with PICRUSt showed that the immune system and repair functions were decreased in SAP. The potential links of gut dysbiosis to immunosuppression and inflammation may contribute to the development of infection post-stroke. Additionally, using multivariate regression analysis, the MDI remained an independent risk factor for SAP and demonstrated good performance in discriminating between the SAP and non-SAP groups (AUCs 75.0% and 80.0%) and increased the predictive performance of A2DS2 score for SAP in two independent cohorts, yielding a potential predictive tool that uses the gut microbiota to detect patients at high risk of SAP. Furthermore, the dysbiosis index exhibited improved sensitivity and specificity compared with those of the use of a single taxon, which suggests that changes in the microbial community rather than individual taxa contribute to SAP development.

Some SCFA-producing taxa, especially *Roseburia*, were depleted in patients with SAP in the two patient cohorts in our study. These results were supported by previous data indicating depletion of SCFA-producing bacteria in patients with infection compared with those without infection after stroke (Haak et al., 2020). Interestingly, significant associations were observed between *Roseburia* and illness status in this study. Decreased gut *Roseburia* abundance was related to systemic inflammation, as reflected by the CRP level, and disease severity, as reflected by discharge and 90-day mRS scores. In multivariate analysis, *Roseburia* remained a protective factor against SAP in both patient cohorts. Additionally, we observed a close relationship between *Roseburia* abundance and SAP severity, as reflected by the PSI and qSOFA scores (Shah et al., 2010; Seymour et al., 2016). These results further underscore the potential roles of this organism in resistance against pneumonia post-stroke. *Roseburia* has been shown to improve the gut ecosystem, prevent leaky gut and exhibit an anti-inflammatory pattern (Hoffmann et al., 2016; Zhu et al., 2018; Seo et al., 2020). Supplementation with *Roseburia* species increased the butyrate level in the dysbiotic bacterial community of patients with Crohn’s disease and improved epithelial barrier function in an *in vitro* model (Hoffmann et al., 2016). *Roseburia* also exhibited an anti-inflammatory pattern (Zhu et al., 2018; Seo et al., 2020) given that it increased anti-inflammatory cytokine IL-22 production and decreased the production of the pro-inflammatory cytokines IFNγ and IL-17 in mono-associated mice (Zhu et al., 2018). Furthermore, *Roseburia* flagellin has been shown to promote gut barrier integrity through upregulation of the expression of the tight junction protein occludin and to help restore the gut microbiota through elevated expression of IL-22 and REG3g (Seo et al., 2020). These above results highlighted that narrow-spectrum therapeutics targeting *Roseburia* might restore intestinal health, attenuate local and systemic inflammation and strengthen epithelial barrier function, which may contribute to the development of personalized therapy for SAP after ischemic stroke. Unexpectedly, some probiotics [the order *Lactobacillales*] were found to be enriched in the SAP patient group. This result can likely be attributed to the controversial role of taxa. Compared with healthy controls, *Lactobacillus* (belongs to *Lactobacillales*) was increased in stroke patients in our previous study (Yin et al., 2015). For the species of *Lactobacillus*, Kazuo Yamashiro et al. (2017) reported that ischemic stroke was associated with increased *Lactobacillus ruminis* and decreased *Lactobacillus sakei* subgroup; and *Lactobacillus ruminis* was positively correlated with system inflammation in these stroke patients. In this study, we observed that there is a higher abundance trend in gut *Lactobacillus* in SAP patients than that of non-SAP patients (Mann Whitney test, P = 0.076, **Figure S6A**); and patients with severe pneumonia (High-PSI group) had higher abundance of gut *Lactobacillus* compared with those in patients with mild pneumonia (Low-PSI group, Mann Whitney test, P = 0.081, **Figure S6B**), indicating the potential relationship between gut *Lactobacillus* and SAP. However, as the 16S rRNA sequence cannot definitively assign identity at the species or strain level, further exploration of the microbiome for SAP will require targeted sequencing methods, ideally with functional metagenomics.

The fecal concentrations of SCFAs in patients with SAP were significantly reduced compared to those in patients without SAP. SCFAs have shown benefits in immunomodulation in both local and distant organs, including the brain and lung (Haak et al., 2018; Antunes et al., 2019; Tan et al., 2020). As an inhibitor of histone deacetylases and mTOR signalling in circulating leukocytes, butyrate directly supports antimicrobial resistance against respiratory pathogens *in vivo* and improves the antimicrobial activity of monocytes and macrophages *in vitro* (Haak et al., 2018; Antunes et al., 2019). In virus-infected mice, microbiota-derived acetate were shown to activate GPR43 on pulmonary epithelial cells, leading to reduced virus-induced cytotoxicity and promoting antiviral effects through the IFN-β response (Antunes et al., 2019). These results suggested that the decrease in SCFA levels observed in the present study may be conducive to sustained inflammation in SAP patients. However, although these metabolites play an important immunomodulatory role in the host, it should be noted that these SCFA-producing bacteria are also capable of directly contributing to resistance against infection by other microbiome constituents and their components, such as the flagellin of *Roseburia* (Seo et al., 2020). Restoration of healthy microbes or SCFA levels can potentially represent a future treatment.

As this was an observational pilot study, some limitations should be mentioned. First, this study did not aim to reveal the precise signaling mechanisms through which the gut microbiota interacts with lung infections post-stroke but rather to elucidate the superficial layer of *in vivo* brain-gut-lung communication, whereby gut dysbiosis with enriched opportunistic pathogens and depleted *Roseburia* post-stroke is a potential risk factor for SAP. Stroke severity, dysphagia and age may promote disruption of the gut microbial profiles in these patients. However, the associations between gut dysbiosis and SAP remained significant after adjustment for age, stroke severity and dysphagia, underscoring the potential risk of gut microbiota dysbiosis at the early acute stage post-stroke for the development of SAP. These results should be interpreted with caution until additional advanced data are acquired to clarify the underlying mechanisms. The next goal in our subsequent studies is to restore the disrupted gut microbiota post-stroke by depleting opportunistic pathogens or enriching *Roseburia* and SCFAs in a mouse model with the hope of attaining associated clinical benefits and explaining the causal relationship in the brain-gut-lung axis. Second, the number of fecal samples and enrolled patients was relatively modest. In this study, we recruited two patient cohorts to confirm the stability of differences in taxa associated with SAP, used the larger patient cohort as the training cohort and further assessed the microbiota-derived metabolites (i.e., SCFAs) and intestinal permeability, with enhanced insights gained from the analyses, possibly increasing the accuracy and credibility. Larger patient cohort should be conducted to reinforce these findings. Finally, the single fecal sample from each patient studied here could not provide a dynamic view of the microbiota and the effects of antibiotic treatment. We believe that the gut flora and SCFAs may change along with the recovery or deterioration of the disease. Longitudinal analyses should be considered as a subject of our future studies.

## Summary

In summary, this study provides the first *in vivo* evidence that patients with *Roseburia* depletion and opportunistic pathogen enrichment in the gut microbiota are at high risk of SAP, which is consistent with the reduced SCFA levels in these patients. These results suggest that larger prospective studies should be undertaken to monitor the microbiome (especially *Roseburia* depletion) of patients with a high risk of SAP. Furthermore, new therapeutic interventions (e.g., bacteriophage therapy) targeting gut bacteria may represent a potential option to prevent SAP and improve the outcomes in these patients.

## Data Availability Statement

The datasets presented in this study can be found in online repositories. The names of the repository/repositories and accession number(s) can be found below: The BioProject database (http://www.ncbi.nlm.nih.gov/bioproject/746092) with accession number PRJNA746092.

## Ethics Statement

The studies involving human participants were reviewed and approved by The Ethical Committee of Southern Medical University. The patients/participants provided their written informed consent to participate in this study.

## Author Contributions

Among the authors in the list, G-HX, JY, YH, and H-WZ designed the study. G-HX, Q-HW, JY, M-SZ, and YH performed and supervised the human experiments. G-HX, H-DW, and M-SZ performed and analyzed all the data. G-HX wrote the manuscript. G-HX, M-SZ, Q-HW, JY, H-WZ, and YH conceived the study, supervised the participants, and revised the manuscript. All authors contributed to the article and approved the submitted version.

## Funding

This study was supported by the National Natural Science Foundation of China (NO. NSFC81870936), the Clinical Research Start up Program of Southern Medical University by High-level University Construction Funding of Guangdong Provincial Department of Education (NO. LC2016PY025) and Clinical Research Program of Nanfang Hospital, Southern Medical University (2018CR024).

## Conflict of Interest

The authors declare that the research was conducted in the absence of any commercial or financial relationships that could be construed as a potential conflict of interest.

## Publisher’s Note

All claims expressed in this article are solely those of the authors and do not necessarily represent those of their affiliated organizations, or those of the publisher, the editors and the reviewers. Any product that may be evaluated in this article, or claim that may be made by its manufacturer, is not guaranteed or endorsed by the publisher.
