# Supplementary material for: Dysbiosis of Gut Microbiota Is an Independent Risk Factor of Stroke-Associated Pneumonia: A Chinese Pilot Study
Source: Front Cell Infect Microbiol. 2021 Aug 3;11:715475. doi: 10.3389/fcimb.2021.715475 (PMC8369370; doi:10.3389/fcimb.2021.715475)
Supplement: Supplementary file 1 [file Table_1.docx]

**SUPPLEMENTAL MATERIAL**

**Tables**

**Table I** Baseline Characteristics of Patients in the validation cohort.

|  | Validation cohort (n=144) | | |
| --- | --- | --- | --- |
|  | Non-SAP  (n=116) | SAP (n=28) | P value |
| Age, y, mean (SD） | 58.9 (12.7) | 68.3 (11.5) | <0.001 |
| Sex, male (%) | 88 (75.9) | 21 (75.0) | 0.924 |
| Hypertension (%) | 86 (74.1) | 24 (86.7) | 0.195 |
| Diabetes mellitus (%) | 45 (38.8) | 15 (53.6) | 0.115 |
| Hyperlipidemia (%) | 54 (46.5) | 10 (35.7) | 0.300 |
| Atrial fibrillation (%) | 7 (6.0) | 1 (3.6) | 0.959 |
| Smoking (%) | 42 (36.2) | 10 (35.7) | 0.961 |
| Stroke history (%) | 26 (22.4) | 14 (50.0) | 0.003 |
| Intial NIHSS score [IQR] | 3.0 [2.0–5.0] | 9.5 [6.0–13.0] | <0.001 |
| Dysphagia (%) | 4 (3.4) | 16 (57.1) | <0.001 |
| A2DS2 score [IQR] | 1 [1–4] | 4 [4–6] | <0.001 |
| WBC, × 10^9^/L | 8.2 [6.6–9.7] | 10.9 [8.7–12.8] | <0.001 |
| Neutrophil, × 10^9^/L | 4.7 [3.7–5.9] | 6.9 [4.7–8.9] | <0.001 |
| Lymphocyte,× 10^9^/L | 1.8 [1.5-2.4] | 1.9 [1.6-2.7] | 0.279 |
| NLR | 2.5 [1.8-3.4] | 3.5 [2.0-4.7] | 0.016 |

The A2DS2 score includes age (1 point for ≥75 years), AF (1 point), dysphagia (2 points), male sex (1 point), and stroke severity (0 point for NIHSS score 0–4, 3 points for NIHSS score 5–15, and 5 points for NIHSS score ≥16). IQR, interquartile range; NIHSS, National Institutes of Health Stroke Scale; SAP, stroke-associated pneumonia. WBC, white blood cell; NLR, neutrophil-to-lymphocyte ratio; CRP, C-reactive protein.

**Table II Relative abundance of selected microbial taxa in patients with and without SAP in the validation cohort.**

|  | Relative abundance (%) | | | Univariate | | Multivariate | |
| --- | --- | --- | --- | --- | --- | --- | --- |
| Taxa | SAP | non-  SAP | P value | OR (95% CI) | AUC | aOR* (95% CI) | aOR† (95% CI) |
| Phylum Proteobacteria | 16.54 | 7.71 | 0.003 | 4.68 (1.65-13.26) | 0.681 | NA | NA |
| Class Gammaproteobacrteria | 11.64 | 4.15 | 0.001 | 3.55 (1.61-7.85) | 0.698 | NA | NA |
| Class Bacilli | 0.90 | 0.24 | 0.014 | 2.08 (1.19-3.66) | 0.649 | 4.42(1.12-17.50) | 4.86 (1.76-13.45) |
| Order Lactobacillales | 0.68 | 0.13 | 0.001 | 1.92 (1.15-3.20) | 0.709 | 2.59(1.02-6.58) | 2.48 (1.19-5.14) |
| Order Enterobacteriales | 11.55 | 3.21 | 0.001 | 3.37 (1.60-7.10) | 0.707 | NA | NA |
| Family Enterococcaceae | 0.006 | 0.000 | 0.035 | 1.31 (1.04-1.64) | 0.611 | NA | NA |
| Family Porphyromonadaceae | 3.03 | 2.04 | 0.279 | NA | 0.566 | NA | NA |
| Family Enterobacteriaceae | 11.55 | 3.21 | 0.001 | 3.37 (1.60-7.10) | 0.707 | NA | NA |
| Genus *Erwinia* | NA | NA | NA | NA | NA | NA | NA |
| Genus *Enterococcus* | 0.006 | 0.00 | 0.035 | 1.31 (1.04-1.64) | 0.612 | NA | NA |
| Genus *Bilophila* | 0.250 | 0.288 | 0.688 | NA | 0.524 | NA | NA |
| Genus *Parabacteroides* | 2.98 | 2.04 | 0.377 | NA | 0.554 | NA | NA |
| Genus *Roseburia* | 0.013 | 0.125 | <0.001 | 0.70 (0.55-0.88) | 0.758 | 0.44 (0.23-0.85) | 0.53 (0.33-0.84) |
| Microbial Dysbiosis Index  (MDI)‡ | 3.7 | 2.4 | <0.001 | 1.52 (1.21-1.90) | 0.800 | 2.22 (1.15-4.26) | 1.97 (1.25-3.09) |

The A2DS2 score includes age (1 point for ≥75 years (1 point) , dysphagia (2 points), male sex (1 point), and stroke severity (0 point for NIHSS score 0-4, 3 points for NIHSS score 5-15, 5 points for NIHSS score ≥16). WBC indicates white blood cell; IQR, interquartile range; NIHSS, National Institutes of Health Stroke Scale; OR, odds ratio; and SAP, stroke-associated pneumonia.

*Adjusted with P <0.10 in univariate analysis(age, hyperlipidemia, AF, dysphagia, white blood cell, neutrophil, lymphocyte, NLR and initial NIHSS score).

†Adjusted with P<0.10 in the univariate analysis and A2DS2(hyperlipidemia, white blood cell, neutrophil, lymphocyte, NLR and A2DS2 score).

‡Index of Microbial dysbiosis (showed as median) , not relative abundance.

**Table III** Baseline characteristics of patients with SAP and non-SAP subjects in the age- and NIHSS- matched dysphagia subgroup in the training cohort.

| Parameters | non-SAP (n=25) | SAP (n=25) | P value |
| --- | --- | --- | --- |
| Age, y, mean (SD） | 61 (12) | 62 (14) | 0.652 |
| Sex, male (%) | 8 (32.0) | 16 (64.0) | 0.024 |
| Hypertension (%) | 16 (64.0) | 19 (73.1) | 0.355 |
| Diabetes mellitus (%) | 8 (32.0) | 6 (24.0) | 0.529 |
| Hyperlipidemia (%) | 2 (8.0) | 0 (0) | 0.470 |
| Atrial fibrillation (%) | 3 (12.0) | 13 (52.0) | 0.002 |
| Smoking (%) | 7 (28.0) | 6 (23.1) | 0.747 |
| Stroke history (%) | 4 (16.0) | 4 (16.0) | 1.000 |
| Intial NIHSS score [IQR] | 8.0 [6.0 –13.5] | 11 [4.5 –15.0] | 0.641 |
| A2DS2 score [IQR] | 5 [4–5] | 5 [4–6] | 0.060 |
| WBC, × 10^9^/L | 8.0 [6.5–10.6] | 8.8 [7.7–12.4] | 0.059 |
| Neutrophil, × 10^9^/L | 5.1 [3.9–7.2] | 6.2 [5.0–9.6] | 0.017 |
| Lymphocyte,× 10^9^/L | 1.7 [1.4–2.4] | 1.5 [0.9–2.0] | 0.090 |
| NLR | 3.1 [2.3–4.0] | 5.0 [3.4–7.2] | 0.006 |
| CRP | 3.1 [1.2–10.1] | 8.2 [2.7–42.5] | 0.016 |

The A2DS2 score includes age (1 point for ≥75 years), atrial fibrillation (1 point), dysphagia (2 points), male sex (1 point), and stroke severity (0 point for NIHSS score 0–4, 3 points for NIHSS score 5–15, and 5 points for NIHSS score ≥16). IQR, interquartile range; NIHSS, National Institutes of Health Stroke Scale; SAP, stroke-associated pneumonia; WBC, white blood cell; NLR, neutrophil-to-lymphocyte ratio; CRP, C-reactive protein.

**Table IV** Outcomes of Patients With or Without SAP in the training cohort.

|  | SAP | non-SAP | P Value | Multivariate * |
| --- | --- | --- | --- | --- |
|  |  |  |  | aOR† (95% CI) |
| Length of stay (LOS, days) | 15.5 [12.0–22.0] | 9.0 [7.0–12.0] | <0.001 | ... |
| Discharge NIHSS score | 9.5 [2.5–13.0] | 2.0 [0–4.0] | <0.001 | ... |
| Gastrointestinal Hemorrhage (GH） | 13 (25.0) | 5 (3.7) | <0.001 | 8.73 (2.93 - 26.02) |
| 30-day mortality, % | 9 (17.3) | 2 (1.5) | <0.001 | 7.17 (1.22 - 42.30) |
| 90-day mRS score, % |  |  | <0.001 | ... |
| Mild (0-2) | 14 (26.9) | 119 (87.5) |  | ... |
| Severe (3-6) | 38 (73.1) | 17 (12.5) |  | 7.22 (2.90 - 17.97) |

* Multivariate analysis model adjusted for age, NIHSS and SAP.

† Adjusted odds ratio of SAP.

**Figures**


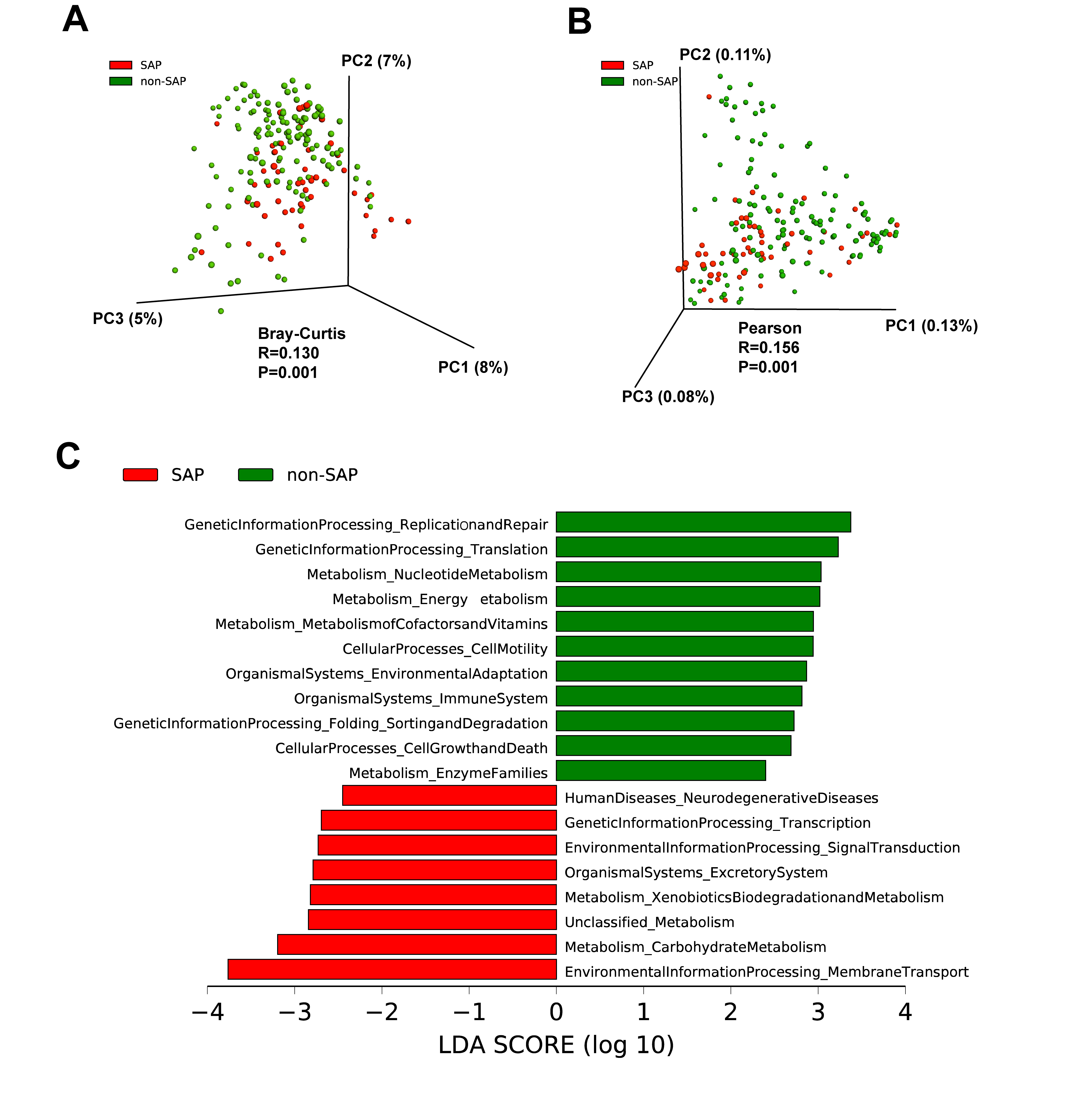


Figure I: The gut microbiota composition of patients with SAP was significantly different from that of non-SAP patients in the training cohort. (A,B) Principal coordinate analysis (PCoA) plot of the Bray-Curtis (A) distance and Pearson distance (B). (C) The PICRUSt algorithm identified the pathway or mechanisms associated with SAP.


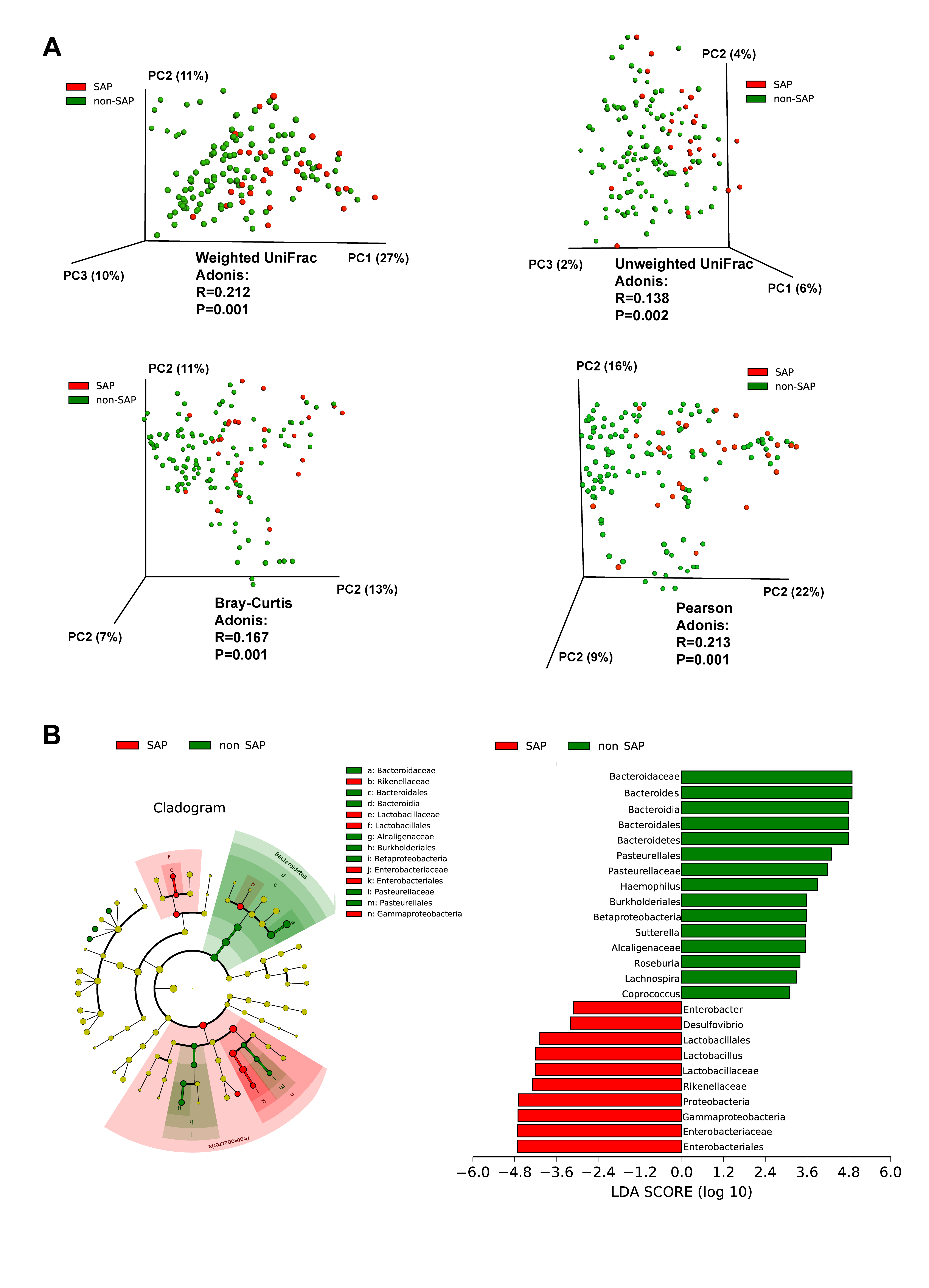


**Figure II**: Comparison of the microbial communities of the SAP and non-SAP groups in the validation cohort. (A) PCoA plot with Weighted UniFrac distance, Unweighted UniFrac distance, Bray-Curtis distance and Pearson distance analyses demonstrating that the bacterial communities were significantly different between the SAP and non-SAP group. (B) LEfSe identified the most differentially abundant taxon between the two groups. Decreased *Roseburia* and enriched pathogens, including *Proteobacteria*, *Enterobacteriaceae* and *Enterococcus,* were in the SAP group compared with those in the non-SAP group.


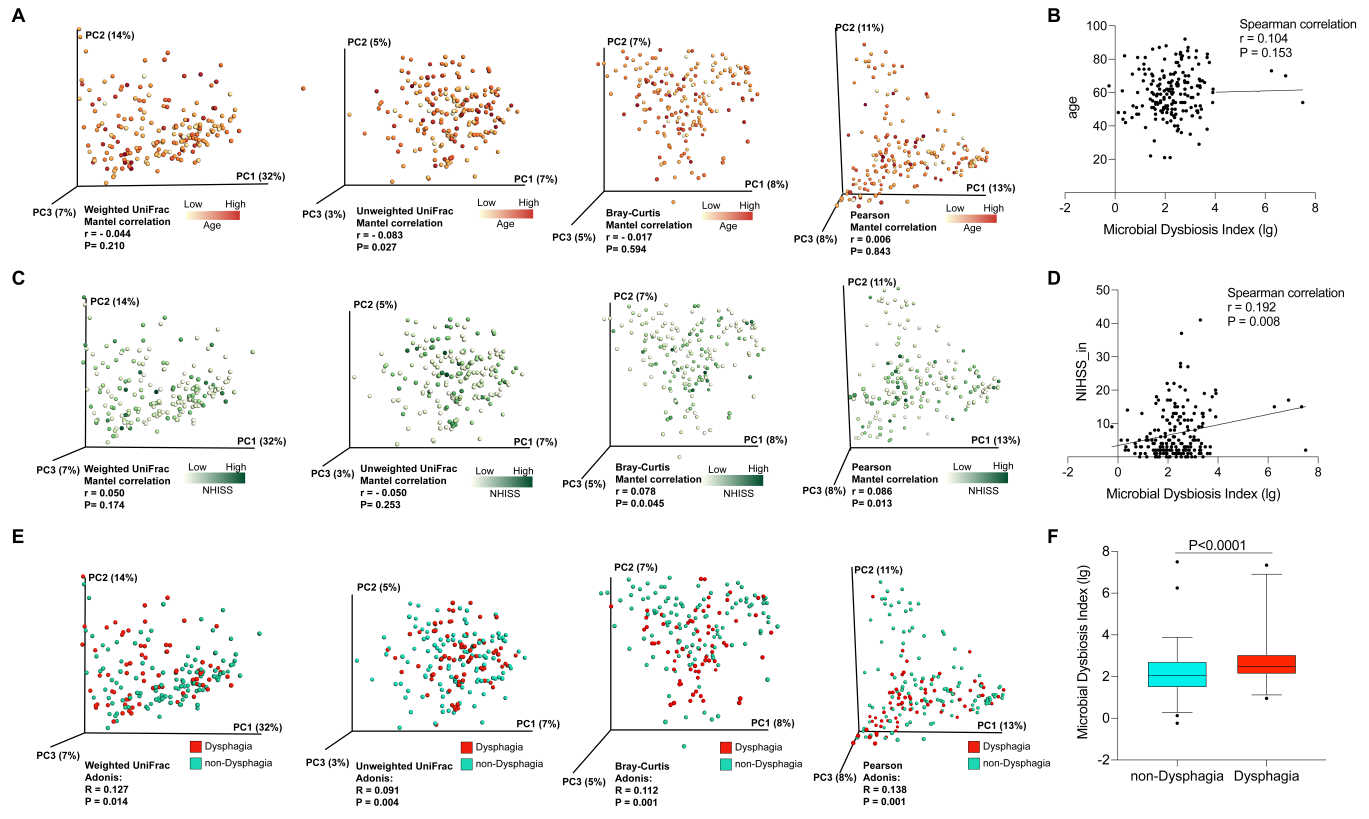


**Figure III**: PCoA plots of the unweighted, weighted UniFrac, Bray-Curtis and Pearson distances in the full sample set (discovery cohort). Samples were coloured by increasing age (A), increasing initial NIHSS score (C). Mantel correlations controlled with 10^4^ permutations were used to compare distances. Spearman’s Correlation of MDI with age (B) or NIHSS (D).

Samples were coloured by dysphagia (E). Box plot showing the MDI in the dysphagia and non-dysphagia group (F).

**
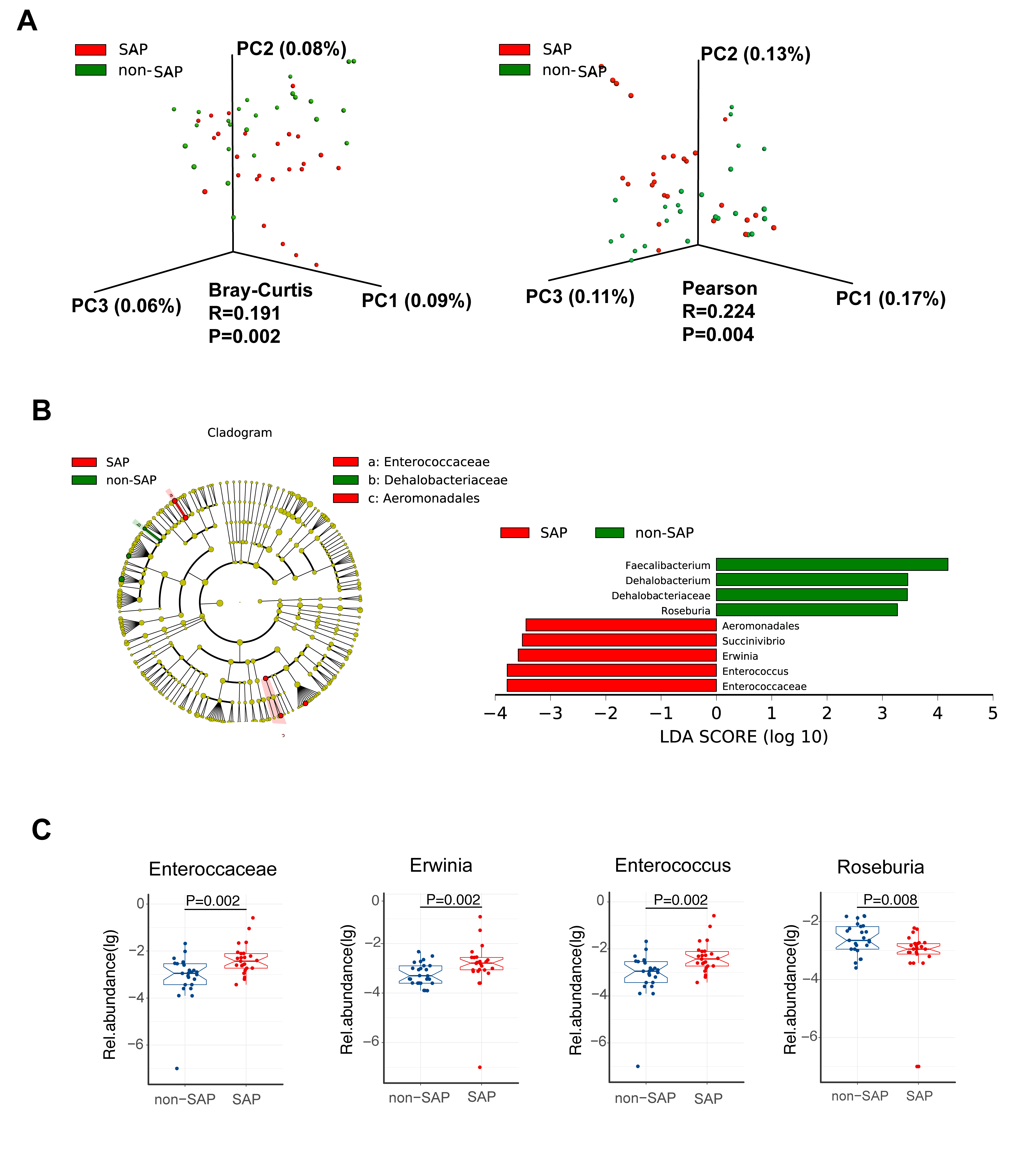
Figure IV**: Comparison of the microbial communities of the SAP and non-SAP groups in the age- and NIHSS- matched subset of the training cohort. (A) PCoA plot with Bray-Curtis and Pearson distance analyses demonstrating that the bacterial communities were significantly different between the SAP and non-SAP group. (B) LEfSe identified the most differentially abundant taxon between the two groups. *Roseburia* was constantly enriched in non-SAP group and opportunistic pathogens, i.e., *Enterococcaceae* and *Enterococcus*, were enriched in SAP group. (C) Relative abundance of the selected taxa differed in the SAP and non-SAP groups. Mann-Whitney *U* test. PC, principal coordinate analysis (PCoA).


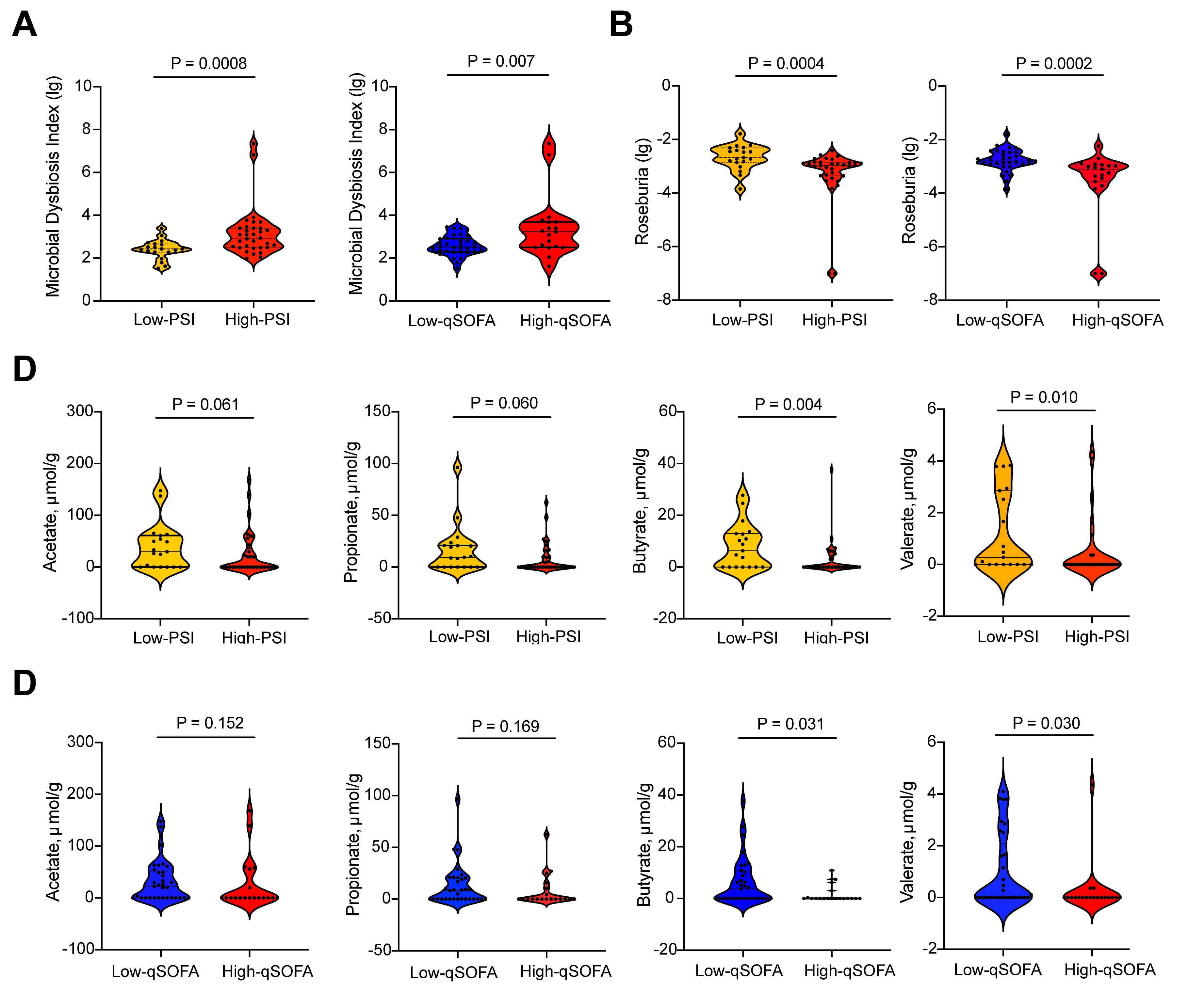


**Figure V**: Differences in the (A) Microbial dysbiosis index (MDI), (B) *Roseburia* and (C, D) SCFA levels according to the pneumonia severity. The high pneumonia severity index (PSI) group (n=33) had significantly (A, left) higher MDI and decreased (B, left) *Roseburia*, (c) butyrate and valerate than the low score group (n=19). Significant higher MDI (A, right), decreased gut *Roseburia* (B, right), butyrate and valerate (D) were observed in the high quick sepsis-related organ failure assessment (qSOFA) group (n=20) than the low qSOFA group (n=32). PSI, pneumonia severity index; qSOFA, quick sepsis-related organ failure assessment.


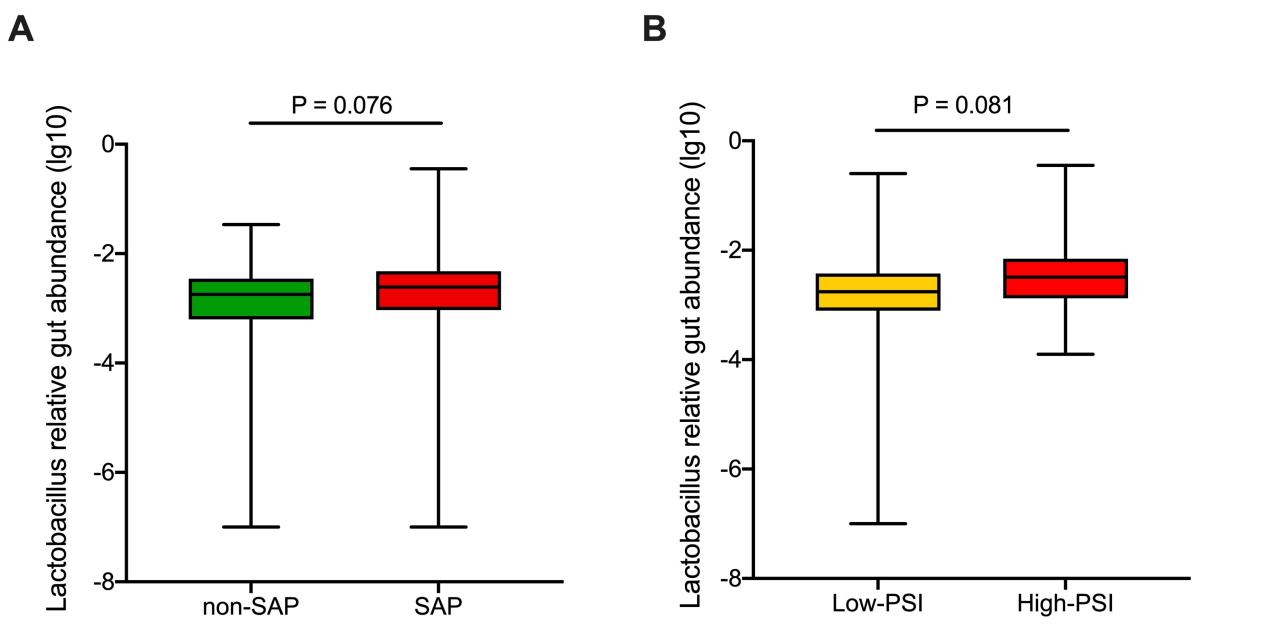


**Figure VI**: Relative abundance of *Lactobacillus* in stroke patients. (**A**) The SAP group have a higher abundance trend in gut *Lactobacillus* than that in non-SAP group. (**B**) The high pneumonia severity index (PSI) group had a higher abundance of gut *Lactobacillus* than the low score group.
